# Protein Subcellular Localization with Gaussian Kernel Discriminant Analysis and Its Kernel Parameter Selection

**DOI:** 10.3390/ijms18122718

**Published:** 2017-12-15

**Authors:** Shunfang Wang, Bing Nie, Kun Yue, Yu Fei, Wenjia Li, Dongshu Xu

**Affiliations:** 1 Department of Computer Science and Engineering, School of Information Science and Engineering, Yunnan University, Kunming 650504, China; bingn2017@gmail.com (B.N.); woshiwenzi666@gmail.com (W.L.); qq78316519@gmail.com (D.X.); 2 School of Statistics and Mathematics, Yunnan University of Finance and Economics, Kunming 650221, China

**Keywords:** protein subcellular localization, kernel parameter selection, kernel discriminant analysis (KDA), Gaussian kernel function, dimension reduction

## Abstract

Kernel discriminant analysis (KDA) is a dimension reduction and classification algorithm based on nonlinear kernel trick, which can be novelly used to treat high-dimensional and complex biological data before undergoing classification processes such as protein subcellular localization. Kernel parameters make a great impact on the performance of the KDA model. Specifically, for KDA with the popular Gaussian kernel, to select the scale parameter is still a challenging problem. Thus, this paper introduces the KDA method and proposes a new method for Gaussian kernel parameter selection depending on the fact that the differences between reconstruction errors of edge normal samples and those of interior normal samples should be maximized for certain suitable kernel parameters. Experiments with various standard data sets of protein subcellular localization show that the overall accuracy of protein classification prediction with KDA is much higher than that without KDA. Meanwhile, the kernel parameter of KDA has a great impact on the efficiency, and the proposed method can produce an optimum parameter, which makes the new algorithm not only perform as effectively as the traditional ones, but also reduce the computational time and thus improve efficiency.

## 1. Introduction

Some proteins can only play the role in one specific place in the cell while others can play the role in several places in the cell [1]. Generally, a protein can function correctly only when it is localized to a correct subcellular location [2]. Therefore, protein subcellular localization prediction is an important research area of proteomics. It is helpful to predict protein function as well as to understand the interaction and regulation mechanism of proteins [3]. Now, many methods have been used to predict protein subcellular location, such as green fluorescent protein labeling [4], mass spectrometry [5], and so on. However, these traditional experimental methods usually have many technical limitations, resulting in high cost of time and money. Thus, prediction of protein subcellular location based on machine learning has become a focus research in bioinformatics [6,7,8].

When we use the methods of machine learning to predict protein subcellular location, we must extract features of protein sequences. We can get some vectors after feature extraction, and then we use the classifier to process these vectors. However, these vectors are usually complex due to their high dimensionality and nonlinear property. In order to improve the prediction accuracy of protein subcellular location, an appropriate nonlinear method for reducing data dimension should be used before classification. Kernel discriminant analysis (KDA) [9] is a nonlinear reductive dimension algorithm based on kernel trick that has been used in many fields such as facial recognition and fingerprint identification. The KDA method not only reduces data dimensionality but also makes use of the classification information. This paper newly introduces the KDA method to predict protein subcellular location. The algorithm of KDA first maps sample data to a high-dimensional feature space by a kernel function, and then executes linear discriminant analysis (LDA) in the high-dimensional feature space [10], which indicates that kernel parameter selection will significantly affect the algorithm performance.

There are some classical algorithms used to select the parameter of kernel function, such as genetic algorithm, grid searching algorithm, and so on. These methods have high calculation precision but large amounts of calculation. In an effort to reduce computational complexity, recently, Xiao et al. proposed a method based on reconstruction errors of samples and used it to select the parameters of Gaussian kernel principal component analysis (KPCA) for novelty detection [11]. Their methods are applied into the toy data sets and UCI (University of CaliforniaIrvine) benchmark data sets to demonstrate the correctness of the algorithm. However, their innovation in the KPCA method aims at dimensional reduction rather than discriminant analysis, which leads to unsatisfied classification prediction accuracy. Thus, it is necessary to improve the efficiency of the method in [11] especially for some complex data such as biological data. 

In this paper, an improved algorithm of selecting parameters of Gaussian kernel in KDA is proposed to analyze complex protein data and predict subcellular location. By maximizing the differences of reconstruction errors between edge normal samples and interior normal samples, the proposed method not only shows the same effect as the traditional grid-searching method, but also reduces the computational time and improves efficiency.

## 2. Results and Discussion

In this section, the proposed method (in Section 3.4) and the grid-searching algorithm (in Section 4.4) are both applied to predict protein subcellular localization. We use two standard data sets as the experimental data. The two used feature expressions are generated from PSSM (position specific scoring matrix) [12], which are the PsePSSM (pseudo-position specific scoring matrix) [12] and the PSSM-S (AAO + PSSM-AAO + PSSM-SAC + PSSM-SD = PSSM-S) [13]. Here AAO means consensus sequence-based occurrence, PSSM-AAO means evolutionary-based occurrence or semi-occurrence of PSSM, PSSM-SD is segmented distribution of PSSM and PSSM-SAC is segmented auto covariance of PSSM. The k-nearest neighbors (KNN) is used as the classifier in which Euclidean distance is adopted for the distance between samples. The flow of experiments is as follows.

- First, for each standard data set, we use the PsePSSM algorithm and the PSSM-S algorithm to extract features, respectively. Then totally we obtain four sample sets, which are GN-1000 (Gram-negative with PsePSSM which contains 1000 features), GN-220 (Gram-negative with PSSM-S which contains 220 features), GP-1000 (Gram-positive with PsePSSM which contains 1000 features) and GP-220 (Gram-positive with PsePSSM which contains 220 features).
- Second, we use the proposed method to select the optimum kernel parameter for the Gaussian KDA model and then use KDA to reduce the dimension of sample sets. The same procedure is also carried out for the traditional grid-searching method to form a comparison with the proposed method.
- Finally, we use the KNN algorithm to classify the reduced dimensional sample sets and use some criterions to evaluate the results and give the comparison results.

Some detailed information in experiments is as follows. For every sample set, we choose the class that contains the most samples to form the training set [8]. Let S=[0.1,0.2,0.3,0.4,1,2,3,4] be a candidate set of the Gaussian kernel parameter, which is proposed at random. When we use the KDA algorithm to reduce dimension, the number of retained eigenvectors must be less than or equal to C−1 (C is the number of classes). Therefore, for sample sets GN-1000 and GN-220, the number of retained eigenvectors, which is denoted as d, can be from 1 to 7. For the sample sets GP-1000 and GP-220, d can be 1, 2, and 3. As far as the parameter u is concerned, when it is 5–8% of the average number of samples, good classification can be achieved [14]. Besides, we demonstrate the robustness of the proposed method with the variation of u in Section 2.2. So here we simply pick a general value for u, say 8. To sum up, in the following experiments, when certain parameters need to be fixed, their default values are as follows. The value of d is 7 for sample sets GN-1000 and GN-220, and 3 for GP-1000 and GP-220; the value of u is 8 and the k value in KNN classifier is 20.

### 2.1. The Comparison Results of the Overall Accuracy

#### 2.1.1. The Accuracy Comparison between the Proposed Method and the Grid-Searching Method

**Figure 1:**
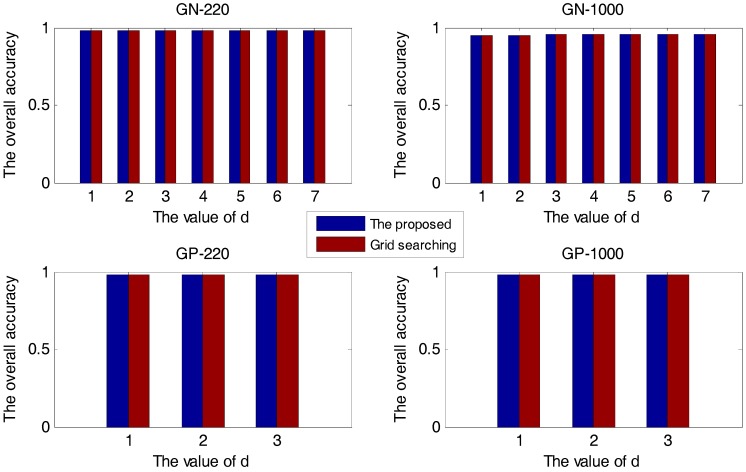
The overall accuracy versus d for four sample sets.

In this section, first, the proposed method and the grid-searching method are respectively used in the prediction of protein subcellular localization with different d values. The experimental results are presented in Figure 1.

In Figure 1, all four sample sets suggest that when we use the KDA algorithm to reduce dimension, the larger the number of retained eigenvectors, the higher the accuracy. The overall accuracy of the proposed method is always the same as that of the grid-searching method, no matter which value of d. The proposed method is effective for selecting the optimal Gaussian kernel parameter.

**Figure 2:**
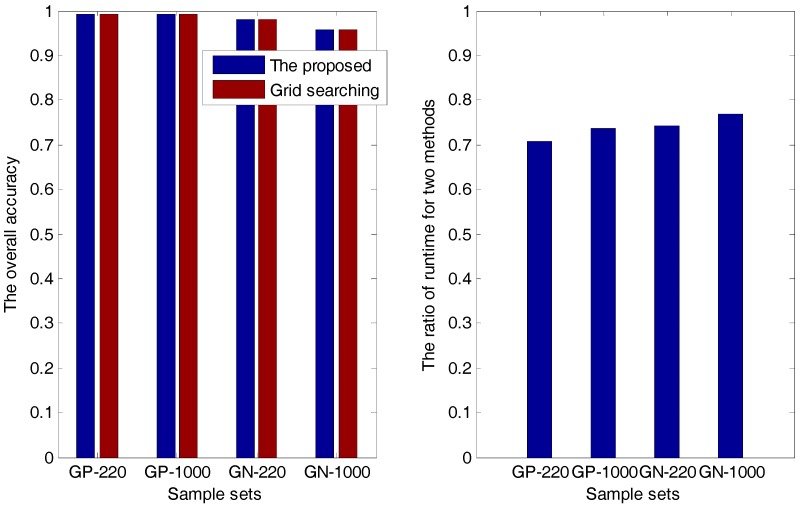
The overall accuracy and the ratio of runtime for two methods.

Then, in the analyses and experiments, we find that superiority of the proposed method is the low runtime, which is demonstrated in Table 1 and Figure 2.

In Table 1, $t_{1}$ and $t_{2}$ are the runtimes of the proposed method and the grid-searching method, respectively. The overall accuracy and the ratio of $t_{1}$ and $t_{2}$ are presented in both Table 1 and Figure 2, from which we can see that for each sample set, the accuracy of the proposed method is always the same as that of the grid-searching method; meanwhile, the runtime of the former is about 70–80% of that of the latter, indicating that the proposed method has a higher efficiency than the grid-searching method.

#### 2.1.2. The Comparison between Methods with and without KDA

**Figure 3:**
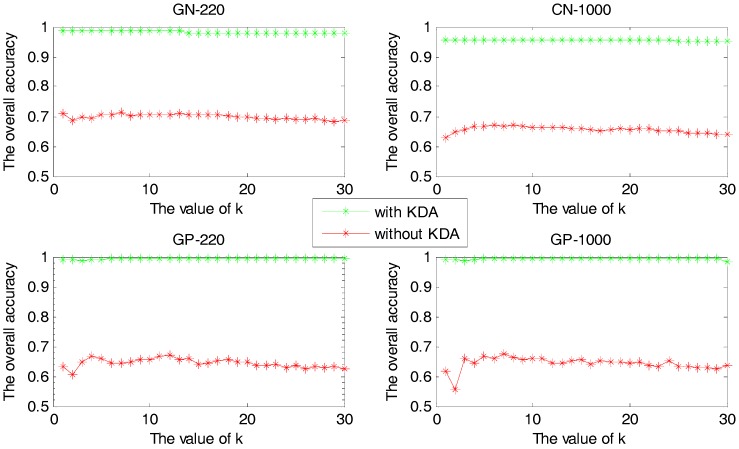
The overall accuracy versus k value with or without KDA algorithm.

In this experiment, we compare the overall accuracies between the cases of using KDA algorithm or not, with k values of the KNN classifier varying from 1 to 30. The experimental results are shown in Figure 3. 

For each sample set, Figure 3 shows that the accuracy with KDA algorithm to reduce dimension is higher than that of without it. However, the kernel parameter has a great impact on the efficiency of the KDA algorithm, and the proposed method can be used to select the optimum parameter that makes the KDA perform perfect. Therefore, accuracy can be improved by using the proposed method to predict the protein subcellular localization.

### 2.2. The Robustness of the Proposed Method

**Figure 4:**
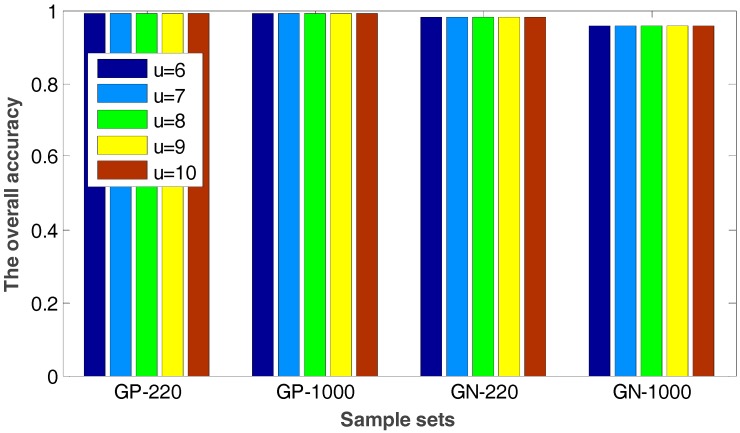
The overall accuracy for four sample sets with different u values.

In the proposed method, the value of u will have an impact on the radius value of neighborhood so that it can affect the number of the selected internal and edge samples. Figure 4 shows the experimental results when the value of u ranges from 6 to 10, in which the overall accuracies of the proposed method and the grid-searching method are given.

It is easily seen from Figure 4 that the accuracy keeps invariable with different u values. The number of the selected internal and edge samples has little effect on the performance of the proposed method. Therefore, the method proposed in this paper has a good robustness.

### 2.3. Evaluating the Proposed Method with Some Regular Evaluation Criterions

In this subsection, we compute the values of some regular evaluation criterions with the proposed method for two standard data sets, which is show in Table 2 and Table 3, respectively. In Table 3, “-” means an infinity value, corresponding to the cases when the denominator is 0 in MCC.

Table 2 and Table 3 show that the values of the evaluation criterion are close to 1 for the proposed method. Then the selection of the kernel parameter using the proposed method will benefit the protein subcellular localization.

## 3. Methods

### 3.1. Protein Subcellular Localization Prediction Based on KDA

**Figure 5:**
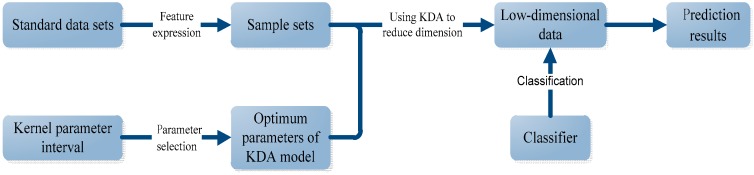
The flow of protein subcellular localization.

To improve the localization prediction accuracy, it is necessary to reduce dimension of high-dimensional protein data before subcellular classification. The flow of protein subcellular localization prediction is presented in Figure 5.

As shown in Figure 5, first, for a standard data set, some features of protein sequences such as PSSM-based expressions are extracted to form the sample sets. The specific feature expressions used in this paper are discussed in Section 4.2. Second, the kernel parameter is selected in an interval based on the sample sets to reach its optimal value in KDA model. Third, with this optimal value, we used the KDA to realize the dimension reduction of the sample sets. Lastly, the low dimensional data is treated by certain classifier to realize the classification and the final prediction. 

In the whole process of Figure 5, dimension reduction with KDA is very important, in which the kernel selection is a key step and constructs the research focus of this paper. Kernel selection includes the choice of the type of kernel function and the choice of the kernel parameters. In this paper, Gaussian kernel function is adopted for KDA because of its good nature, learning performance, and catholicity. So, the emphasis of this study is to decide the scale parameter of the Gaussian kernel, which plays an important role in the process of dimensionality reduction and has a great influence on prediction results. We put forward a method for selecting the optimum Gaussian kernel parameter with the starting point of reconstruction error idea in [15].

### 3.2. Algorithm Principle

Kernel method constructs a subspace in the feature space by the kernel trick, which makes normal samples locate in or nearby this subspace, while novel samples are far from it. The reconstruction error is the distance of a sample from the feature space to the subspace [11], so the reconstruction errors of normal samples should be different from those of the novel samples. In this paper, we use the Gaussian KDA as the descending algorithms. Since the values of the reconstruction errors are influenced by the Gaussian kernel parameters, the reconstruction errors of normal samples should be differentiated from those of the novel samples by suitable parameters [11].

In the input space, we usually call the samples on the boundary as edge samples, and call those within the boundary as internal samples [16,17]. The edge samples are much closer to novel samples than the internal samples, while the internal samples are much closer to normal states than the edge samples [11]. We usually use the internal samples as the normal samples and use the edge samples as the novel samples, since there are no novel samples in data sets. Therefore, the principle is that the optimal kernel parameter makes the reconstruction errors have a reasonable difference between the internal samples and the edge samples.

### 3.3. Kernel Discriminant Analysis (KDA) and Its Reconstruction Error

KDA is an algorithm by applying kernel trick into linear discriminant analysis (LDA). LDA is an algorithm of linear dimensionality reduction together with classifying discrimination, which aims to find a direction that maximizes the between-class scatter while minimizing the within-class scatter [18]. In order to extend the LDA theory to the nonlinear data, Mika et al. proposed the KDA algorithm, which makes the nonlinear data linearly separable in a much higher dimensional feature space than before [9]. The principle of the KDA algorithm is shown as follows. 

Suppose the N samples in X can be divided into C classes and the ith class contains $N_{i}$ samples satisfying $N=\sum_{i=1}^{C}N_{i}$. The between-class scatter matrix $S_{b}^{\phi }$ and the within-class scatter matrix $n_{n}^{\phi }$ of X are defined in the following equations, respectively:

(1) $$S_{b}^{\phi }=\sum_{i=1}^{C}N_{i}\left(m_{i}^{\phi }-m^{\phi }\right)\;\left(m_{i}^{\phi }-m^{\phi }\right){\;}^{T}$$

(2) $$S_{w}^{\phi }=\sum_{i=1}^{C}\sum_{j=1}^{N_{i}}\left[\phi \left(x_{j}^{i}\right)-m_{i}^{\phi }\right]\;\left[\phi \left(x_{j}^{i}\right)-m_{i}^{\phi }\right]{}^{T}$$

where $m_{i}^{\phi }=\frac{1}{N_{i}}\sum_{j=1}^{N_{i}}\phi \left(x_{j}^{i}\right)$ is the mean vector of the ith class, and $m^{\phi }=\frac{1}{N}\sum_{i=1}^{N}\phi \left(x_{i}\right)$ is the total mean of X. To find the optimal linear discriminant, we need to maximize J(W) as follows:

(3) $$max\;J\left(W\right)=\frac{W^{T}S_{b}^{\phi }W}{W^{T}S_{w}^{\phi }W}$$

where $W={\left[w_{1},w_{2},\cdots ,w_{d}\right]}^{T}\left(1\leq d\leq C-1\right)$ is a projection matrix, and $w_{k}\left(k=1,2,\cdots ,d\right)$ is a column vector with N elements. Through certain algebra, it can be deduced that W is made up of the eigenvectors corresponding to the top d eigenvalues of $S_{w}^{\phi }{}^{-1}S_{b}^{\phi }$. Also, the projection vector $w_{k}$ can be represented by a linear combination of the samples in the feature space:

(4) $$w_{k}=\sum_{j=1}^{N}a_{j}^{k}j\left(x_{j}\right)$$

where $a_{j}^{k}$ is a real coefficient. The projection of the sample X onto $w_{k}$ is given by:

(5) $$w_{k}^{T}\times \phi \left(x\right)=\sum_{i=1}^{N}a_{i}^{k}K\left(x,x_{j}\right)$$

Let $a=\left[a^{1},a^{2},\cdots ,a^{d}\right]{}^{T}$ be the coefficient matrix where $a^{k}=\left[a_{1}^{k},a_{2}^{k},\cdots ,a_{N}^{k}\right]{}^{T}$ is the coefficient vector. Combining Equations (1)–(5), we can obtain the linear discriminant by maximizing the function J(a):

(6) $$max\;J\left(a\right)=\frac{a^{T}\tilde{M}a}{a^{T}\tilde{L}a}$$

where $\tilde{M}=\sum_{i=1}^{C}N_{i}\left(M_{i}-M\right)\;\left(M_{i}-M\right){}^{T}$, $\tilde{L}=\sum_{i=1}^{C}K_{i}\left(E-\frac{1}{N_{i}}I\right)K_{i}^{T}$, the kth component of the vector $M_{i}$ is ${\left(M_{i}\right)}_{k}=\frac{1}{N_{i}}\sum_{j=1}^{N_{i}}K\left(x_{k},x_{j}^{i}\right)\;\left(k=1,2,\cdots ,N\right)$, the kth component of the vector M is ${\left(M\right)}_{k}=\frac{1}{N}\sum_{j=1}^{N}K\left(x_{k},x_{j}^{i}\right)\;\left(k=1,2,\cdots ,N\right)$, $K_{i}$ is a $N\times N_{i}$ matrix with ${\left(K_{i}\right)}_{mn}=K\left(x_{m},x_{n}^{i}\right)$, E is the $N_{i}\times N_{i}$ identity matrix, and $\frac{1}{N_{i}}I$ is the $N_{i}\times N_{i}$ matrix that all elements are $\frac{1}{N_{i}}$ [9]. Then, the projection matrix a is made up of the eigenvectors corresponding to the top d eigenvalues of ${\tilde{L}}^{-1}\tilde{M}$.

According to the KDA algorithm principle in (3) or (6), besides the Gaussian kernel parameter s, the number of retained eigenvectors d also affects the algorithm performance. Generally, in this paper, the proposed method is mainly used to screen an optimum S under a predetermined d value. 

The Gaussian kernel function is defined as follows:

(7) $$K\left(x_{i},x_{j}\right)=exp(-\frac{{\left\|x_{i}-x_{j}\right\|}^{2}}{\sigma^{2}})$$

where σ is the scale parameter which is generally estimated by s. Note that ${\left\|\phi \left(x\right)\right\|}^{2}=K\left(x,x\right)=1$.

The kernel-based reconstruction error is defined in the following equation:

(8) $$\begin{array}{ll} RE\left(x\right) & ={\left\|\phi \left(x\right)-W\;t\left(x\right)\;\right\|}^{2}={\left\|\phi \left(x\right)\;\right\|}^{2}-{\left\|\;t\left(x\right)\;\right\|}^{2}\\ & =K\left(x,x\right)-{\left\|\;t\left(x\right)\;\right\|}^{2} \end{array}$$

where t(x) is the vector obtained by projecting ϕ(x) onto a projection matrix a. 

### 3.4. The Proposed Method for Selecting the Optimum Gaussian Kernel Parameter

The method of kernel parameter selection relies on the reconstruction errors of the internal samples and the edge samples. Therefore, first we find a method to select the edge samples and the interior samples, then we propose the method for selection of the Gaussian kernel parameter.

#### 3.4.1. The Method for Selecting Internal and Edge Samples

Li and Maguire present a border-edge pattern selection method (BEPS) to select the edge samples based on the local geometric information [16]. Xiao et al. [11] modified the BEPS algorithm so that it can select both the edge samples and internal samples. However, their algorithm has the risk of making all samples in the training set become the edge samples. For example, when all samples are distributed on a spherical surface in a three-dimensional space, every sample in the data set will be selected as the edge samples since its neighbors are all located on one side of its tangent plane. In order to solve this problem, this paper innovatively combines the ideas in [19,20] to select the internal and edge samples, respectively, which is not dependent on the local geometric information. The main principle is that the edge sample is usually surrounded by the samples belonging to other classes while the internal sample is usually surrounded by the samples belonging to its same class. Further, the edge samples are usually far from the centroid of this class, while the internal samples are usually close to the centroid. So, a sample will be selected as the edge sample if it is far from the centroid of this class and there are samples around it that belongs to other classes, otherwise it will be selected as the internal sample.

Specifically, suppose the ith class $X_{i}=\left\{x_{1},x_{2},\cdots ,x_{N_{i}}\right\}$ in the sample set X is picked out as the training set. Denote $c_{i}$ be the centroid of this class:

(9) $$c_{i}=\frac{1}{N_{i}}\sum_{i=1}^{N_{i}}x_{i}$$

We use the median value m of the distances from all samples in a class to its centroid to measure the distance from a sample to the centroid of this class. A sample is conserved to be far from the centroid of this class if the distance from this sample to the centroid is greater than the median value. Otherwise, the sample is considered to be close to the centroid. 

Denote $dist\left(x_{i},x_{j}\right)$ as the distance between any two samples $x_{i}$ and $x_{j}$, and $N_{\varepsilon }\left(x\right)$ as the ε-neighborhood of X:

(10) $$N_{\varepsilon }\left(x\right)=\left\{y|dist\left(x,y\right)\leq \varepsilon ,y\in X_{}\right\}$$

The value of neighborhood ε is given as follows. Let u be a given number which satisfies $0<u<N_{i}$. $Density_{u}\left(X_{i}\right)$ is the mean radius of neighborhood of $X_{i}$ for the given number u:

(11) $$Density_{u}\left(X_{i}\right)=\frac{1}{N_{i}}\sum_{i=1}^{N_{i}}dist_{u}\left(x_{i}\right)$$

where $dist_{u}\left(x_{i}\right)$ is the distance from $x_{i}$ to its uth nearest neighbor. So, $Density_{u}\left(X_{i}\right)$ is used as the value of ε for the training set $X_{i}$. The flow for the selection of the internal and edge samples is shown in Table 4.

In Table 4, a sample X is considered to be the edge one when the distance from X to the centroid is larger than the median m and there are samples in $N_{\varepsilon }\left(x\right)$ belonging to other classes in this case. A sample X is considered to be the internal one when the distance from X to the centroid is less than m and in this case all samples of $N_{\varepsilon }\left(x\right)$ belong to this class.

#### 3.4.2. The Proposed Method

In order to select the optimum kernel parameter, it is necessary to propose a criterion aiming to distinguish reconstruction errors of the edge samples from those of the internal samples. A suitable parameter not only maximizes the difference between reconstruction errors of the internal samples and those of the edge samples, but also minimizes the variance (or standard deviation) of reconstruction errors of the internal samples [11]. According to the rule, an improved objective function is proposed in this paper. The optimal Gaussian kernel parameter S is selected by maximizing this objective function.

(12) $$s=arg\underset{s}{max}f\left(s\right)=arg\;\underset{s}{max}\;\frac{{\left\|RE\left(\Omega_{ed}\right)\;\right\|}_{\infty }-{\left\|RE\left(\Omega_{in}\right)\;\right\|}_{\infty }}{std\left\{RE\left(\Omega_{in}\right)\right\}}$$

where ${\left\|\;\cdot \;\right\|}_{\infty }$ is the infinite norm which computes the maximum absolute component of a vector and std(⋅) is a function of the standard deviation. Note that in the objective function f(s), our key improvement is to use the infinite norm to compute the size of reconstruction error vector since it can lead to a higher accuracy than many other measurements, which has been verified by a series of our experiments. The reason is probably that the maximum component is more reasonable to evaluate the size of a reconstruction error vector than others such as the 1-norm, p-norm (1<p<+∞) and the minimum component of a reconstruction error vector in [11].

According to (8), when the number of retained eigenvectors is determined, we can select the optimum parameter s from a candidate set using the proposed method. The optimum parameter ensures that the Gaussian KDA algorithm performs well in dimensionality reduction, which improves the accuracy of protein subcellular location prediction. The proposed method for selecting the Gaussian kernel parameter can be presented in Table 5. 

As the end of this section, we want to summarize the position of the proposed method in protein subcellular localization once more. First, two kinds of regularization forms of PSSM are used to extract the features in protein amino acid sequences. Then, the KDA method is performed on the extracted features for dimension reduction and discriminant analysis according to the KDA algorithm principle in Section 3.3 with formulas (1)–(6). During the procedure of KDA, the novelty of our work is to give a new method for selecting the Gaussian kernel parameter, which is summarized in Table 5. Finally, we choose the k-nearest neighbors (KNN) as the classifier to cluster the dimension-reduced data after KDA.

## 4. Materials

In this section, we introduce the other processes in Figure 5 except KDA model and its parameter selection, which are necessary materials for the whole experiment. 

### 4.1. Standard Data Sets

In this paper, we use two standard datasets that have been widely used in the literature for Gram-positive and Gram-negative subcellular localizations [13], whose protein sequences all come from the Swiss-Prot database. 

For the Gram-positive bacteria, the standard data set we found in the literature [13,14,21] is publicly available on http://www.csbio.sjtu.edu.cn/bioinf/Gpos-multi/Data.htm. There are 523 locative protein sequences in the data set that are distributed in four different subcellular locations. The number of proteins in each location is given in Table 6.

For the Gram-negative bacteria, the standard data set of subcellular localizations is presented in the literature [13,22], which can be downloaded freely from http://www.csbio.sjtu.edu.cn/bioinf/Gneg-multi/Data.htm. The data set contains 1456 locative protein sequences located in eight different subcellular locations. The number of proteins in each location is shown in Table 7.

### 4.2. Feature Expressions and Sample Sets

In the prediction of protein subcellular localizations with machine learning methods, feature expressions are important information extracted from protein sequences, which have certain proper mathematical algorithms. There are many efficient algorithms used to extract features of protein sequences, in which two of them, PsePSSM [12] and PSSM-S [13], are used in this paper. The two methods rely on the position-specific scoring matrix (PSSM) for benchmarks which is obtained by using the PSI-BLAST algorithm to search the Swiss-Prot database with the parameter E-value of 0.01. The PSSM is defined as follows [12]:

(13) $$P_{PSSM}=\left[\begin{array}{cccc} M_{1\to 1} & M_{1\to 2} & \cdots & M_{1\to 20}\\ M_{2\to 1} & M_{2\to 2} & \cdots & M_{2\to 20}\\ \vdots & \vdots & \vdots & \vdots \\ M_{i\to 1} & M_{i\to 2} & \cdots & M_{i\to 20}\\ \vdots & \vdots & \vdots & \vdots \\ M_{L\to 1} & M_{L\to 2} & \cdots & M_{L\to 20} \end{array}\right]$$

where $M_{i\to j}$ represents the score created in the case when the ith amino acid residue of the protein sequence is transformed to the amino acid type j during the evolutionary process [12]. 

Note that, usually, multiple alignment methods are used to calculate PSSM, whose chief drawback is being time-consuming. The reason why we select PSSM instead of simple multiple alignment in this paper to form the total normalized information content is as follows. First, since our focus is to demonstrate the effectiveness of dimensional reduction algorithm, we need to construct high-dimensional feature expressions such as PsePSSM and PSSM-S, whose dimensions are as high as 1000 and 220, respectively. Second, PSSM has many advantages, such as those described in [23]. As far as the information features are concerned, PSSM has produced the strongest discriminator feature between fold members of protein sequences. Multiple alignment methods are used to calculate PSSM, whose chief drawback is being time-consuming. However, in spite of the time-consuming nature of constructing a PSSM for the new sequence, the extracted feature vectors from PSSM are so informative that are worth the cost of their preparation [23]. Besides, for a new protein sequence, we only need to construct a PSSM for the first time, which could be used repeatedly in the future for producing new normalization forms such as PsePSSM and PSSM-S.

#### 4.2.1. Pseudo Position-Specific Scoring Matrix (PsePSSM)

Let P be a protein sample, whose definition of PsePSSM is given as follows [12]:

(14) $$P_{Pse-PSSM}^{\xi }={\left[{\bar{M}}_{1}{\bar{M}}_{2}\cdots {\bar{M}}_{20}G_{1}^{\xi }G_{2}^{\xi }\cdots G_{20}^{\xi }\right]}^{T}(\xi =0,1,2,\cdots ,49)$$

(15) $${\bar{M}}_{j}=\frac{1}{L}\sum_{i=1}^{L}M_{i\to j}(j=1,2,\cdots ,20)$$

(16) $$G_{j}^{\xi }=\frac{1}{L-\xi }{\sum_{i=1}^{L-\xi }\left[M_{i\to j}-M_{\left(i+\xi \right)\to j}\right]}^{2}(j=1,2,\cdots ,20;\xi <L)$$

where L is the length of P, $G_{j}^{\xi }$ is the correlation factor by coupling the ξ-most contiguous scores [22]. According to the definition of PsePSSM, a protein sequence can be represented by a 1000-dimensional vector.

#### 4.2.2. PSSM-S

Dehzangi et al. [13] put forward a new feature extraction method, PSSM-S, which combines four components: AAO, PSSM-AAO, PSSM-SD, and PSSM-SAC. According to the definition of the PSSM-S, it can be represented a feature vector with 220 (20 + 20 + 80 + 100) elements.

#### 4.2.3. Sample Sets

For the two benchmark data, PsePSSM and PSSM-S are used to extract features, respectively. Finally we get four experimental sample sets GN-1000, GN-220, GP-1000 and GP-220, shown in Table 8. 

### 4.3. Evaluation Criterion

To evaluate the performance of the proposed method, we use Jackknife cross-validation, which has been widely used to predict protein subcellular localization [13]. The Jackknife test is the most objective and rigorous cross-validation procedure in examining the accuracy of a predictor, which has been used increasingly by investigators to test the power of various predictors [24,25]. In the Jackknife test (also known as leave-one-out cross-validation), every protein is removed one-by-one from the training dataset, and the predictor is trained by the remaining proteins. The isolated protein is then tested by the trained predictor [26]. Let x be a sample set with N samples. For each sample, it will be used as the test data, and the remaining N−1 samples will be used to construct the training set [27]. In addition, we use some criterion to assess the experimental results, defined as follows [12]:

(17) $$MCC\left(k\right)=\frac{TP_{k}\times TN_{k}-FN_{k}\times FP_{k}}{\sqrt{\left(TP_{k}+FN_{k}\right)\;\left(TP_{k}+FP_{k}\right)\;\left(TN_{k}+FP_{k}\right)\;\left(TN_{k}+FN_{k}\right)}}\times 100\%$$

(18) $$Sen\left(k\right)=\frac{TP_{k}}{TP_{k}+FN_{k}}\times 100\%$$

(19) $$Spe\left(k\right)=\frac{TN_{k}}{TP_{k}+FP_{k}}\times 100\%$$

(20) $$Q=\frac{\sum_{k=1}^{C}TP_{k}}{N}\times 100\%$$

where TP is the number of true positive, TN is the number of true negative, FP is the number of false positive, and FN is the number of false negative [12]. The value of MCC (Matthews coefficient correlation) varies between −1 and 1, indicating when the classification effect goes from a bad to a good one. The values of Specificity (Spe), sensitivity (Sen), and the overall accuracy (Q) all vary between 0 and 1, and the classification effect is better when their values are closer to 1, while the classification effect is worse when their values are closer to 0 [13].

### 4.4. The Grid Searching Method Used as Contrast

In this section, we introduce a normal algorithm for searching S, the grid-searching algorithm, which is used as a contrast with the proposed algorithm in Section 3.4. 

The grid-searching method is usually used to select the optimum parameter, whose steps are as follows for the candidate parameter set S [28].

- Compute the kernel matrix k for each parameter $s_{i}\in S,\;i=1,2,\cdots ,m$.
- Use the Gaussian KDA to reduce the dimension of K.
- Use the KNN algorithm to classify the reduced dimensional samples.
- Calculate the classification accuracy.
- Repeat the above four steps until all parameters in S have been traversed. The parameter corresponding to the highest classification accuracy is selected as the optimum parameter.

## 5. Conclusions

Biological data is usually high-dimensional. As a result, it is necessary to reduce dimension to improve the accuracy of the protein subcellular localization prediction. The kernel discriminant analysis (KDA) based on Gaussian kernel function is a suitable algorithm for dimensional reduction in such applications. As is known to all, the selection of a kernel parameter affects the performance of KDA, and thus it is important to choose the proper parameter that makes this algorithm perform well. To handle this problem, we propose a method of the optimum kernel parameter selection, which relies on reconstruction error [15]. Firstly, we use a method to select the edge and internal samples of the training set. Secondly, we compute the reconstruction errors of the selected samples. Finally, we select the optimum kernel parameter that makes the objective function maximum.

The proposed method is applied to the prediction of protein subcellular locations for Gram-negative bacteria and Gram-positive bacteria. Compared with the grid-searching method, the proposed method gives higher efficiency and performance.

Since the performance of the proposed method largely depends on the selection of the internal and edge samples, in the future study, researchers may pay more attention to select more representative internal and edge samples from the biological data set to improve the prediction accuracy of protein subcellular localization. Besides this, it is also meaningful to research how to further improve the proposed method to make it suitable for selecting parameters of other kernels.

## Figures and Tables

**Table 1:** The overall accuracy and the ratio of runtime for two methods.

Sample Sets	Overall Accuracy		Ratio ($t_{1}/t_{2}$)
GP-220 (PSSM-S)	The proposed method	0.9924	0.7087
	Grid searching method	0.9924	
GP-1000 (PsePSSM)	The proposed method	0.9924	0.7362
	Grid searching method	0.9924	
GN-220 (PSSM-S)	The proposed method	0.9801	0.7416
	Grid searching method	0.9801	
GN-1000 (PsePSSM)	The proposed method	0.9574	0.7687
	Grid searching method	0.9574	

**Table 2:** The values of evaluation criterion with the proposed method for the Gram-positive.

Sample Set	Protein Subcellular Locations			
	Cell Membrane	Cell Wall	Cytoplasm	Extracell
**Sensitivity**				
GP-220	1	0.9444	0.9904	0.9919
GP-1000	0.9943	0.9444	1	0.9837
**Specificity**				
GP-220	0.9943	1	1	09950
GP-1000	0.9971	1	0.9937	0.9925
**Matthews coefficient correlation (MCC)**				
GP-220	0.9914	0.9709	0.9920	0.9841
GP-1000	0.9914	0.9709	0.9921	0.9840
**Overall accuracy (Q)**				
GP-220	0.9924			
GP-1000	0.9924			

**Table 3:** The values of evaluation criterion with the proposed method for the Gram-negative.

Sample Set	Protein Subcellular Locations							
	(1)	(2)	(3)	(4)	(5)	(6)	(7)	(8)
**Sensitivity**								
GN-220	1	0.9699	1	0	0.9982	0	0.9677	1
GN-1000	1	0.9323	1	0	0.9659	0	0.9516	0.9556
**Specificity**								
GN-220	0.9924	0.9902	1	1	0.9978	1	1	0.9953
GN-1000	0.9608	0.9872	1	1	0.9967	1	1	0.9992
**Matthews coefficient correlation (**MCC**)**								
GN-220	0.9866	0.9324	1	-	0.9956	-	0.9823	0.9814
GN-1000	0.9346	0.8957	1	-	0.9681	-	0.9733	0.9712
**Overall accuracy (Q)**								
GN-220	0.9801							
GN-1000	0.9574							

(1) Cytoplasm, (2) Extracell, (3) Fimbrium, (4) Flagellum, (5) Inner membrane, (6) Nucleoid, (7) Outer membrane, (8) Periplasm.

**Table 4:** The Selection of Internal and Edge Samples.

Input: $X=\left\{X_{1},X_{2},\cdots ,X_{C}\right\}$, the training set $X_{i}=\left\{x_{1},x_{2},\cdots ,x_{N_{i}}\right\}$ (1≤i≤C).
1. Calculate the radius of neighborhood ε using Equation (11).
2. Calculate the centroid $c_{i}$ of the i^t^^h^ class according to Equation (9).
3. Calculate the distances $dist_{j}(j=1,2,\cdots ,N_{i})$ from all samples in training set to $c_{i}$, respectively, and the median value m of them.
4. For each training sample $x_{j}$ of the set $X_{i}$ Calculate the $N_{\varepsilon }\left(x_{j}\right)$ according to Equation (10). If $dist_{j}>m$ and there are samples in $N_{\varepsilon }\left(x_{j}\right)$ belonging to other classes, $x_{j}$ is selected as an edge sample. If $dist_{j}<m$ and no sample in $N_{\varepsilon }\left(x_{j}\right)$ belongs to other classes, $x_{j}$ is selected as an internal sample.
**Output: the selected internal sample set** $\Omega_{in}$**, the selected edge sample set** $\Omega_{ed}$**.**

**Table 5:** The Method for Selecting the Gaussian KDA Parameter.

Input: A reasonable candidate set $S=\left\{s_{1},s_{2},\cdots ,s_{m}\right\}$ for Gaussian kernel parameter, $X=\left\{X_{1},X_{2},\cdots ,X_{C}\right\}$, the training set $X_{i}=\left\{x_{1},x_{2},\cdots ,x_{N_{i}}\right\}$ (1≤i≤C), the number of retained eigenvectors d.
1. Get the internal sample set $\Omega_{in}$ and the edge sample set $\Omega_{ed}$ from the training set $X_{i}$ using Algorithm 1.
2. For each parameter $s_{i}\in S,\;i=1,2,\cdots ,m$ Calculate the kernel matrix K using Equation (7). Reduce dimension of the K using the Gaussian KDA algorithm. Calculate $RE\left(\Omega_{ed}\right)$ and $RE\left(\Omega_{in}\right)$ using Equation (8). Calculate the value of objective function $f\left(s_{i}\right)$ using Equation (12).
3. Select the optimum parameter $s=arg\underset{s_{i}\in S}{max}f\left(s_{i}\right)$
Output: the optimum Gaussian kernel parameter S.

**Table 6:** The name and the size of each location for the Gram-positive data set.

No.	Subcellular Localization	Number of Proteins
1	cell membrane	174
2	cell wall	18
3	cytoplasm	208
4	extracell	123

**Table 7:** The name and the size of each location for the Gram-negative data set.

No.	Subcellular Localization	Number of Proteins
1	cytoplasm	410
2	extracell	133
3	fimbrium	32
4	flagellum	12
5	inner membrane	557
6	nucleoid	8
7	outer membrane	124
8	periplasm	180

**Table 8:** Sample sets.

Sample Sets	Benchmarks for Subcellular Locations	Extraction Feature Method	The Number of Classes	The Dimension of Feature Vector	The Number of Samples
GN-1000	Gram-negative	PsePSSM	8	1000	1456
GN-220	Gram-negative	PSSM-S	8	220	1456
GP-1000	Gram-positive	PsePSSM	4	1000	523
GP-220	Gram-positive	PSSM-S	4	220	523
